# Osimertinib in advanced EGFR-T790M mutation-positive non-small cell lung cancer patients treated within the Special Use Medication Program in Spain: OSIREX-Spanish Lung Cancer Group

**DOI:** 10.1186/s12885-021-07922-5

**Published:** 2021-03-06

**Authors:** Mariano Provencio, Josefa Terrasa, Pilar Garrido, Rosario García Campelo, Francisco Aparisi, Pilar Diz, David Aguiar, Carlos García-Giron, Julia Hidalgo, Carlos Aguado, Jorge García González, Emilio Esteban, Lorenzo Gómez-Aldavarí, Teresa Moran, Oscar Juan, Luís Enrique Chara, Juan L. Marti, Rafael López Castro, Ana Laura Ortega, Elia Martínez Moreno, Juan Coves, Ana M. Sánchez Peña, Joaquim Bosch-Barrera, Amparo Sánchez Gastaldo, Natalia Fernández Núñez, Edel del Barco, Manuel Cobo, Dolores Isla, Margarita Majem, Fátima Navarro, Virginia Calvo

**Affiliations:** 1 Medical Oncology Department, Hospital Universitario Puerta de Hierro, Majadahonda, Madrid Spain; 2 Health Research Institute, Hospital Universitario Puerta de Hierro, Majadahonda, Madrid Spain; 3 Universidad Autónoma de Madrid, Madrid, Spain; 4 Medical Oncology Department, Hospital Universitari Son Espases, Islas Balears, Palma de Mallorca, Spain; 5 Medical Oncology Department, IRYCIS Hospital Universitario Ramón y Cajal, Madrid, Spain; 6 Medical Oncology Department, Hospital Universitario A Coruña, A Coruña, Spain; 7 Instituto de Investigación Biomédica A Coruña INIBIC, A Coruña, Spain; 8 Medical Oncology Department Valencia, Hospital General Universitario de Valencia, Madrid, Spain; 9 Medical Oncology Department León, Complejo Asistencial Universitario de León, Madrid, Spain; 10 Medical Oncology Department, Hospital Universitario de Gran Canaria Dr. Negrín, Las Palmas de GC, Spain; 11 Medical Oncology Department, Hospital Universitario De Burgos, Burgos, Spain; 12 Medical Oncology Department, Hospital Lluís Alcanyis, Xátiva, Valencia Spain; 13 Medical Oncology Department, Hospital Clínico San Carlos, Madrid, Spain; 14 Medical Oncology Department Santiago de Compostela, Hospital Clínico Universitario de Santiago, Madrid, Spain; 15 Medical Oncology Department, Hospital Universitario Central de Asturias, Oviedo, Spain; 16 Medical Oncology Department, Complejo Hospitalario Universitario de Albacete, Albacete, Spain; 17 Institut Català d’Oncologia Badalona, Medical Oncology Department, Badalona Barcelona, Spain; 18 Hospital Universitari Germans Trias i Pujol, Barcelona, Badalona Spain; 19 Department of Medicine, Universitat Autònoma de Barcelona, Barcelona, Spain; 20 Badalona Applied Research Group in Oncology, Barcelona, Spain; 21 Fundació Germans Trias i Pujol, Barcelona, Spain; 22 Medical Oncology Department, Hospital Universitari i Politècnic La Fe, Valencia, Spain; 23 Medical Oncology Department, Hospital Universitario de Guadalajara, Guadalajara, Spain; 24 Medical Oncology Department, Hospital General Universitario de Alicante, Alicante, Spain; 25 Medical Oncology Department, Hospital Clínico Universitario de Valladolid, Valladolid, Spain; 26 Medical Oncology Department, Complejo Hospitalario de Jaen, Jaen, Spain; 27 Medical Oncology Department, Hospital Virgen de la Salud, Toledo, Spain; 28 Medical Oncology Department, Hospital Son Llàtzer, Palma de Mallorca, Spain; 29 Medical Oncology Department, Hospital Universitario de Getafe, Getafe, Madrid Spain; 30 Department of Oncology, Catalan Institute of Oncology. Dr. Josep Trueta University Hospital, Girona, Spain; 31 Medical Oncology Department, Hospital Universitario Virgen del Rocío, Sevilla, Spain; 32 Medical Oncology Department, Hospital Universitario Lucus Augusti, Lugo, Spain; 33 Medical Oncology Department, Hospital Universitario de Salamanca, Salamanca, Spain; 34 Hospitales Universitarios Regional y Virgen de la Victoria, IBIMA, Unidad de Gestión Clínica Intercentros de Oncología Médica, Málaga, Spain; 35 Medical Oncology Department, Hospital Universitario Lozano Blesa, Zaragoza, Aragón Spain; 36 Medical Oncology Department, Hospital de la Santa Creu i Sant Pau, Barcelona, Spain; 37 Medical Oncology Department, Hospital Universitario Príncipe de Asturias, Alcalá de Henares, Madrid Spain

**Keywords:** Osimertinib, Real-world data, EGFR-activating mutations, T790M EGFR mutation, Second line, Non-small cell lung cancer

## Abstract

**Background:**

AURA study reported 61% objective response rate and progression-free survival of 9.6 months with osimertinib in patients with EGFR/T790M+ non-small cell lung cancer. Due to lack of real-world data, we proposed this study to describe the experience with osimertinib in Spain.

**Methods:**

Post-authorization, non-interventional Special Use Medication Program, multicenter, retrospective study in advanced EGFR/T790M+ non-small cell lung cancer. One hundred-fifty five patients were enrolled (August 2016–December 2018) from 30 sites. Primary objective: progression-free survival. Secondary objectives: toxicity profile, objective response rate, and use of health service resources.

**Results:**

70% women, median age 66. 63.9% were non-smokers and 99% had adenocarcinoma. Most patients had received at least one prior treatment (97%), 91.7% had received previous EGFR-tyrosine kinase inhibitors and 2.8% osimertinib as first-line treatment. At data cutoff, median follow-up was 11.8 months. One hundred-fifty five patients were evaluable for response, 1.3% complete response, 40.6% partial response, 31% stable disease and 11.6% disease progression. Objective response rate was 42%. Median progression-free survival was 9.4 months. Of the 155 patients who received treatment, 76 (49%) did not reported any adverse event, 51% presented some adverse event, most of which were grade 1 or 2. The resource cost study indicates early use is warranted.

**Conclusion:**

This study to assess the real-world clinical impact of osimertinib showed high drug activity in pretreated advanced EGFR/T790M+ non-small cell lung cancer, with manageable adverse events.

**Trial registration:**

Clinical trial registration number**:** NCT03790397.

## Background

In Spain occur about 27,351 new lung cancer cases per year and the disease was responsible for 22,896 deaths in 2018 [1, 2]. Most non-small cell lung cancer (NSCLC) patients are diagnosed with unresectable disease and around 40% with advanced disease.

The identification of oncogenic driver alterations that can be targeted by EGFR tyrosine kinase inhibitors (EGFR-TKI: erlotinib, gefitinib, afatinib, dacotinib) for EGFR mutated tumors has improved median progression-free survival (PFS), overall response rates and quality of life in metastatic NSCLC patients, making EGFR TKIs the first-line treatment of choice in patients with EGFR mutation (EGFRm) advanced NSCLC [3]. Unfortunately, however, even these patients eventually develop resistance.

Multiple resistance mechanisms have been observed, including the EGFR Thr790Met (T790M) resistance mutation, MET amplification, HER2 amplification and small-cell histological transformation, among others. EGFR T790M mutations have been detected in 48–62% of patients that develop resistance to EGFR-TKIs [4, 5].

Until recently, treatment options in the post-EGFR-TKI second-line setting were limited, with low rates of response to platinum-based doublet chemotherapies [6–8].

Osimertinib (AZD9291) is a potent oral irreversible EGFR-TKI that is selective for both EGFR and T790M resistance mutations with activity in the central nervous system (CNS) [9, 10]. In the phase 1 component of the phase 1/2 AURA trial (ClinicalTrials.gov number, NCT01802632), objective response rate (ORR) for osimertinib in patients with T790M-positive NSCLC was 61% and median PFS was 9.6 months [11].

The publication of the FLAURA study, which obtained a significantly longer PFS and overall survival (OS) than with standard EGFR-TKI in first-line (18.9 months vs. 10.2 months), led to the approval of osimertinib for standard first-line treatment.

Osimertinib has been approved in Europe and commercialized in Spain for the treatment of patients with EGFR T790M-positive (EGFR/T790M+) advanced NSCLC and in first line regardless the T790M mutation status.

Then, just after the ASTRIS study had closed recruitment [12], the Foreign Medication Program was opened. This is a Special Use Medication Program (SUMP) from the Agencia Española de Medicamentos y Productos Sanitarios (AEMPS) to access osimertinib in Spain for the patient population without other treatment options for pretreated patients with EGFR/T790M+ advanced NSCLC.

Based on the lack of real-world results in this setting, and the experience with osimertinib in Spain since 2015, we proposed a retrospective study to describe the experience in terms of efficacy and safety in EGFR/T790M+ NSCLC.

## Methods

### Study population

This was a post-authorization-other-designs, non-interventional, multicenter, retrospective study that in no way interfered with physicians’ normal clinical practice. The study is based on collection of data about those patients treated with osimertinib within the SUMP and therefore did not involve any diagnostic or therapeutic procedures beyond normal clinical practice. The results provide some insights into the clinical efficacy of osimertinib treatment and consumption of hospital primary care resources.

Eligible patients had histologically or cytologically confirmed stage IIIb/IV EGFR/T790M+ NSCLC and had received osimertinib treatment within the osimertinib Spanish Special Use Medication Program (SUMP).

Living patients had to have signed and dated an Independent Ethics Committee/Institutional Review Board (IRB/EC)-approved written informed consent form following regulatory and institutional guidelines. This had to be obtained before the performance of any protocol-related procedures that were not part of normal care.

### Assessment

The primary objective was to estimate PFS to osimertinib. Secondary objectives were to establish osimertinib toxicity profile, estimate duration of response (DOR), ORR by RECIST v1.1 and report on the consumption of hospital and primary care resources.

All clinical efficacy objectives were to be assessed depending on treatment line (first, second, third or further lines).

Date of progression, type of progression, DOR defined as time from documentation of tumor response until disease progression or death, and ORR by RECIST v1.1 were measured.

Adverse events (AEs) related to osimertinib treatment received within the SUMP in Spain were filed in a Remote Data Capture system (RDC)/Case Report Form (CRF). Type and severity of AEs were classified according to the NCI CTCAE Version 5.0. The causal relationship to the study drug was determined by the physician and should be used to assess all AEs. The casual relationship may be either “related” or “not related”.

### Independent ethics committee (IEC) or institutional review board (IRB)

The study was evaluated by the Ethical Committee of Hospital Universitario Puerta de Hierro de Majadahonda, Madrid.

The study was also classified by the Spanish Health Authority as “EPA-OD” (post-authorization study, other designs). The ethical committees of other hospitals also reviewed the study as per local practice. The study was carried out following the Declaration of Helsinki on Good Clinical Practices and Organic Law 3/2018, 5th December on Protection of Personal Data and digital rights that were guaranteed during the study. The study was coordinated and monitored by the Spanish Lung Cancer Group (SLCG) and financed by AstraZeneca following the specifications in the protocol. This funding included the cost of submitting the study for approval to an accredited IEC; submission for classification to the AEMPS; database design, maintenance and management; monitoring activities; statistical analysis and corresponding statistical report.

### Data quality assurance

The application used for data collection had safety margins and internal coherence rules to avoid input of incorrect data or anomalous or incoherent values. Data quality and queries were revised and dealt with by the SLCG Data Management Department. All patient information required was included in a Remote Data Capture system (RDC)/Case Report Form (CRF).

### Statistical analysis

A total of approximately 156 patients were included from several Spanish sites. The observation period was from August 2016 and December 2018.

The following descriptive statistics were used. Frequency statistics for categorical variables: number and percentage. Mean, standard deviation, median (interquartile range) and categorical distribution for quantitative variables (e.g. number of exacerbations, hospital admissions, emergency unit visits, prescriptions, primary care visits). In addition, two-sided 95% confidence intervals (CI) were presented for the specified study outcomes.

PFS was defined from initiation of osimertinib treatment until radiological and/or clinical progression or death from any cause, whichever occurred first. OS was defined from initiation of osimertinib to death from any cause.

Progression was ideally measured by RECIST v 1.1; patients with unknown progression status at time of data collection were censored at the date they were last known to be free of radiological and/or clinical progression. DOR was defined as time from documentation of tumor response to disease progression or death.

PFS and DOR were estimated using Kaplan–Meier analysis.

ORR, defined as percentage of patients achieving complete or partial response, was evaluated based on RECIST v 1.1 response criteria.

Clinical efficacy variables were assessed for patients (squamous vs non-squamous) depending on whether they received first, second, third or subsequent lines of treatment. Safety of osimertinib administration was described by tabulation of the CTCAE version 5.0. No inferential analyses were foreseen. No interim analyses were predefined. All study results were considered exploratory and descriptive by design. All statistical analyses were performed using R software.

### Protocol deviations

There were no major protocol deviations as this was not a clinical trial and data collection was carried out in a timely manner and according to protocol. One hundred and fifty-six patients were included but only 155 were valid since 1 patient was an inclusion error.

## Results

### Patient characteristics

One hundred and fifty-five patients who started osimertinib treatment between August 2016 and December 2018 were analyzed. Table 1 shows the main demographic data. One hundred and fifty-five were T790M positive: 70% (109/155) had EGFR deletion in exon 19, 25% (39/155) exon 21, and 5% (7/155) other previous mutation status. Four patients (2.6%) received osimertinib as first line, 83 (53.5%) as second line, 31 (20.0%) as third line and 37 (23.9%) as the fourth of further lines.

**Table 1:** Patient clinical characteristics

	Total
**Patients**	**155 (100%)**
**Sex**	
Male	47 (30.3%)
Female	108 (69.7%)
**Age**	
Mean (SD)	66.9 (11.4)
Median (Min.-Max.)	67 (37–88)
**Race**	
Caucasian	149 (96.2%)
Latin	5 (3.2%)
African	1 (0.6%)
**Smoking habit**	
Non-smoker (≤100 cigarettes over lifetime)	99 (63.9%)
Former smoker (≥1 year)	41 (26.5%)
Smoker	14 (9.0%)
Not provided	1 (0.6%)
**Performance Status (PS)**	
0	43 (27.7%)
1	75 (48.4%)
2	10 (6.5%)
3	3 (1.9%)
Not reported	24 (15.5%)
**Histology**	
Adenocarcinoma	154 (99.4%)
Undifferentiated	1 (0.6%)
**M staging**	
MX	1 (0.6%)
M0	27 (17.5%)
M1	127 (81.9%)
**Metastatic sites**	
Extra-thoracic adenopathy	16 (10.3%)
Thoracic adenopathy	40 (25.8%)
Meningeal carcinomatosis	2 (1.3%)
Pericardial effusion	7 (4.5%)
Pleural effusion	46 (29.7%)
Liver	19 (12.3%)
Bones	59 (38.1%)
Bilateral lymphangitis	7 (4.5%)
Pleural nodes	29 (18.7%)
Peritoneal	4 (2.6%)
Lung	79 (51.0%)
Central nervous system	22 (14.2%)
Subcutaneous	1 (0.6%)
Suprarenal	9 (5.8%)
Soft tissue	2 (1.3%)
Choroids	2 (1.3%)

Palliative radiotherapy use was also registered. Of the 155 patients included, 73 (47.1%) received radiotherapy treatment during follow up, of whom 60 (38.7%) started radiotherapy before beginning osimertinib treatment, and 13 (8.4%) started radiotherapy after beginning osimertinib treatment.

Regarding chemotherapy, 152 of the 155 patients included (98.1%) received chemotherapy during follow up.

### Efficacy results

According to RECIST v 1.1, best response to osimertinib was distributed thus: 2 (1.3%) patients with complete response (CR), 63 (40.6%) with partial response (PR), 48 (31%) with stable disease (SD), 18 (11.6%) with progression and 24 (15.5%) without registered response. Of the 131 patients with registered response to osimertinib treatment, estimated objective response (PR or CR reached) was 42%. Regarding comparison of response rates between treatment lines when osimertinib was administered, no statistically significant differences were observed among the 75 patients who received osimertinib in first or second line with registered response (of the 35 that responded, 46.7%) and the 56 patients who received osimertinib in third or fourth line with registered response (of the 30 that responded, 53.6%) (*p*-value = 0.482). Table 2 shows the drugs administered as first, second and third lines prior to osimertinib.

**Table 2:** Drugs administered as first, second and third line prior to osimertinib

	***N***	%
**First-line treatment prior to osimertinib**	**151**	**100%**
**Type of treatment**		
**Monotherapy**	**120**	**79.5%**
Gefitinib	51	33.8%
Erlotinib	39	25.8%
Afatinib	24	15.9%
Cisplatin	2	1.3%
Dacomitinib	2	1.3%
Pembrolizumab	1	0.7%
Pemetrexed	1	0.7%
**Combination (2 drugs)**	**27**	**17.9%**
Cisplatin + Pemetrexed	7	4.6%
Cisplatin + Vinorelbine	6	4.0%
Carboplatin + Pemetrexed	5	3.3%
Bevacizumab + Erlotinib	2	1.3%
Carboplatin + Vinorelbine	2	1.3%
Gefitinib + Olaparib	2	1.3%
Carboplatin + Paclitaxel	1	0.7%
Cisplatin + Docetaxel	1	0.7%
Cisplatin + Etoposide VP16	1	0.7%
**Combination (3 drugs)**	**4**	**2.6%**
Bevacizumab + Carboplatin + Paclitaxel	2	1.3%
Bevacizumab + Cisplatin + Pemetrexed	2	1.3%
**Second-line treatment prior to osimertinib**	**68**	**100%**
**Type of treatment**		
**Monotherapy**	**40**	**58.8%**
Erlotinib	19	27.9%
Gefitinib	9	13.2%
Afatinib	7	10.3%
Pemetrexed	2	2.9%
Docetaxel	1	1.5%
Gemcitabine	1	1.5%
Rociletinib	1	1.5%
**Combination (2 drugs)**	**21**	**30.9%**
Carboplatin + Pemetrexed	9	13.2%
Cisplatin + Pemetrexed	7	10.3%
Bevacizumab + Erlotinib	1	1.5%
Carboplatin + Etoposide VP16	1	1.5%
Carboplatin + Paclitaxel	1	1.5%
Gefitinib + Olaparib	1	1.5%
Gefitinib + Pemetrexed	1	1.5%
**Combination (3 drugs)**	**6**	**8.8%**
Bevacizumab + Carboplatin + Paclitaxel*	4	5.9%
Bevacizumab + Cisplatin + Pemetrexed	1	1.5%
Carboplatin + Erlotinib + Pemetrexed	1	1.5%
**Combination (4 drugs)**	**1**	**1.5%**
Atezolizumab + Bevacizumab + Carboplatin + Paclitaxel**	1	1.5%
**Third-line treatment prior to osimertinib**	**37**	**100%**
**Type of treatment**		
**Monotherapy**	**23**	**62.2%**
Erlotinib	7	18.9%
Afatinib	5	13.5%
Gefitinib	3	8.1%
Pemetrexed	3	8.1%
Vinorelbine	2	5.4%
Docetaxel	1	2.7%
Nivolumab	1	2.7%
Rociletinib	1	2.7%
**Combination (2 drugs)**	**12**	**32.4%**
Carboplatin + Pemetrexed	6	16.2%
Docetaxel + Nintedanib	2	5.4%
Afatinib + Cetuximab	1	2.7%
Carboplatin + Paclitaxel	1	2.7%
Cisplatin + Pemetrexed	1	2.7%
Gemcitabine + Paclitaxel	1	2.7%
**Combination (3 drugs)**	**2**	**5.4%**
Atezolizumab + Carboplatin + Paclitaxel	1	2.7%
Carboplatin + Erlotinib + Pemetrexed	1	2.7%

### Adverse events (AEs)

Table 3 shows a summary of AEs on osimertinib classified according to the CTCAEv5.0. One hundred and fifty-five patients received treatment and were included in the study. Of these, 76 (49.0%) did not show AEs; 29 (18.7%) showed AEs with a maximum grade of 1; 32 (20.6%) showed AEs with a maximum grade of 2; 14 (9%) showed AEs with a maximum grade of 3; 2 (1.3%) with a maximum grade of 4; and 2 (1.3%) with a maximum grade of 5.

**Table 3:** Adverse events on treatment with osimertinib

		Total	1	2	3	4	5
**Patient**		**155 (100%)**	–	–	–	–	–
**Without adverse events**		76 (49.0%)	–	–	–	–	–
**With adverse events**		79 (51.0%)	29	32	14	2	2
1. Blood and lymphatic system disorders	Anemia	4 (2.6%)	2	2	0	0	0
2. Cardiac disorders	Chest pain - cardiac	1 (0.6%)	1	0	0	0	0
	Heart failure	1 (0.6%)	0	0	1	0	0
	Sinus bradycardia	1 (0.6%)	1	0	0	0	0
3. Congenital, familial and genetic disorders	Congenital, familial and genetic disorders - Other, specify	1 (0.6%)	0	1	0	0	0
4. Ear and labyrinth disorders	Hearing impaired	1 (0.6%)	0	1	0	0	0
5. Endocrine disorders	Cushingoid	1 (0.6%)	1	0	0	0	0
6. Eye disorders	Dry eye	2 (1.3%)	2	0	0	0	0
	Extraocular muscle paresis	1 (0.6%)	0	1	0	0	0
	Eye disorders - other, specify	1 (0.6%)	0	1	0	0	0
	Keratitis	1 (0.6%)	1	0	0	0	0
	Watering eyes	1 (0.6%)	1	0	0	0	0
7. Gastrointestinal disorders	Abdominal pain	1 (0.6%)	1	0	0	0	0
	Colitis	1 (0.6%)	0	1	0	0	0
	Diarrhea	24 (15.5%)	16	8	0	0	0
	Dry mouth	1 (0.6%)	1	0	0	0	0
	Gastrointestinal disorders - other, specify	4 (2.6%)	1	2	1	0	0
	Gingival pain	1 (0.6%)	0	0	1	0	0
	Mucositis oral	10 (6.5%)	6	3	1	0	0
	Nausea	9 (5.8%)	6	2	1	0	0
	Vomiting	5 (3.2%)	4	1	0	0	0
8. General disorders and administration site conditions	Edema limbs	1 (0.6%)	1	0	0	0	0
	Fatigue	16 (10.3%)	10	2	4	0	0
	General disorders and administration site conditions - other, specify	1 (0.6%)	0	1	0	0	0
	Pain	1 (0.6%)	1	0	0	0	0
11. Infections and infestations	Conjunctivitis	2 (1.3%)	1	1	0	0	0
	Eye infection	1 (0.6%)	0	1	0	0	0
	Fungemia	1 (0.6%)	0	1	0	0	0
	Joint infection	1 (0.6%)	0	0	1	0	0
	Nail infection	1 (0.6%)	1	0	0	0	0
	Paronychia	6 (3.9%)	4	1	1	0	0
	Rash pustular	2 (1.3%)	0	2	0	0	0
13. Investigations	Lipase increased	1 (0.6%)	0	0	1	0	0
	Lymphocyte count decreased	1 (0.6%)	0	0	1	0	0
	Neutrophil count decreased	3 (1.9%)	0	3	0	0	0
	Platelet count decreased	8 (5.2%)	3	4	0	0	1
	Serum amylase increased	1 (0.6%)	1	0	0	0	0
	White blood cell decreased	1 (0.6%)	0	1	0	0	0
14. Metabolism and nutrition disorders	Anorexia	7 (4.5%)	5	0	2	0	0
15. Musculoskeletal and connective tissue disorders	Arthralgia	2 (1.3%)	2	0	0	0	0
	Back pain	1 (0.6%)	1	0	0	0	0
	Bone pain	1 (0.6%)	1	0	0	0	0
	Flank pain	1 (0.6%)	1	0	0	0	0
	Generalized muscle weakness	1 (0.6%)	1	0	0	0	0
17. Nervous system disorders	Cognitive disturbance	1 (0.6%)	0	0	1	0	0
	Dizziness	3 (1.9%)	3	0	0	0	0
	Dysgeusia	2 (1.3%)	2	0	0	0	0
	Headache	1 (0.6%)	1	0	0	0	0
	Paresthesia	2 (1.3%)	1	1	0	0	0
19. Psychiatric disorders	Agitation	1 (0.6%)	1	0	0	0	0
	Anxiety	2 (1.3%)	2	0	0	0	0
	Confusion	1 (0.6%)	1	0	0	0	0
22. Respiratory, thoracic and mediastinal disorders	Cough	1 (0.6%)	0	1	0	0	0
	Dyspnoea	7 (4.5%)	3	2	1	1	0
	Pneumonitis	4 (2.6%)	2	0	0	1	1
	Respiratory, thoracic and mediastinal disorders - other, specify	2 (1.3%)	1	1	0	0	0
23. Skin and subcutaneous tissue disorders	Dry skin	2 (1.3%)	1	1	0	0	0
	Nail changes	1 (0.6%)	1	0	0	0	0
	Palmar-plantar erythrodysesthesia syndrome	2 (1.3%)	0	1	1	0	0
	Pruritus	4 (2.6%)	3	1	0	0	0
	Rash acneiform	12 (7.7%)	9	3	0	0	0
	Rash maculo-papular	3 (1.9%)	2	1	0	0	0
	Skin and subcutaneous tissue disorders - other, specify	11 (7.1%)	7	4	0	0	0
26. Vascular disorders	Thromboembolic event	3 (1.9%)	1	1	1	0	0
	Vascular disorders - other, specify	1 (0.6%)	0	0	1	0	0

Osimertinib dose was adjusted in 26 patients (16%) and was due to toxicity in 18 of these (11.6%). Treatment was discontinued in 4 patients (2.6% of the total treated) due to toxicity.

### Follow up and survival

Patients were monitored from the beginning of osimertinib treatment until time of death, loss to follow up or study closure while they were still alive. Median follow up for all patients was 11.7 months (range 0.4–32.0 months), while the median for those patients that were alive was 14.9 months (range 1.8–32.0 months).

**Fig. 1:**
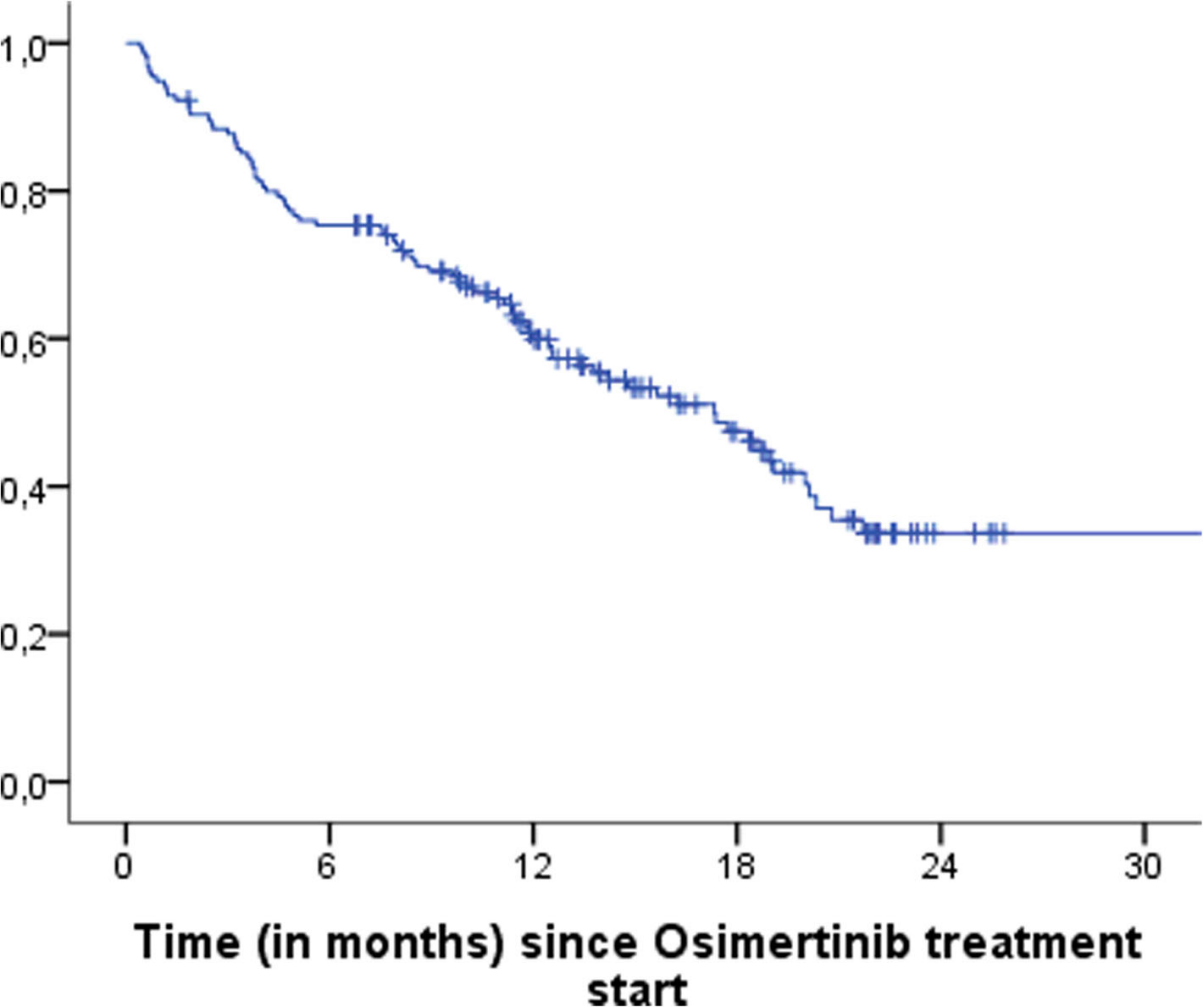
Estimated median OS of patients treated with osimertinib

Of the 155 patients included, 80 (51.6%) died during follow up. Estimated median OS for these patients was 17.3 months (95% CI, 13.4–21.3 months). Figure 1.

Overall survival can be compared depending on the line of treatment patients received. Of the 87 patients who received osimertinib as first (4 patients) or second line (83 patients), 46 died during follow up (52.9%). Of the 68 patients that received osimertinib as third (31 patients), forth (22 patients), fifth (11 patients), sixth (2 patients) or seventh line (2 patients), 34 died during follow up (50.0%). No statistically significant differences are observed between the overall survival curves of these two groups of patients (osimertinib as <2nd line versus >3rd line, *p*-value = 0.392).

**Fig. 2:**
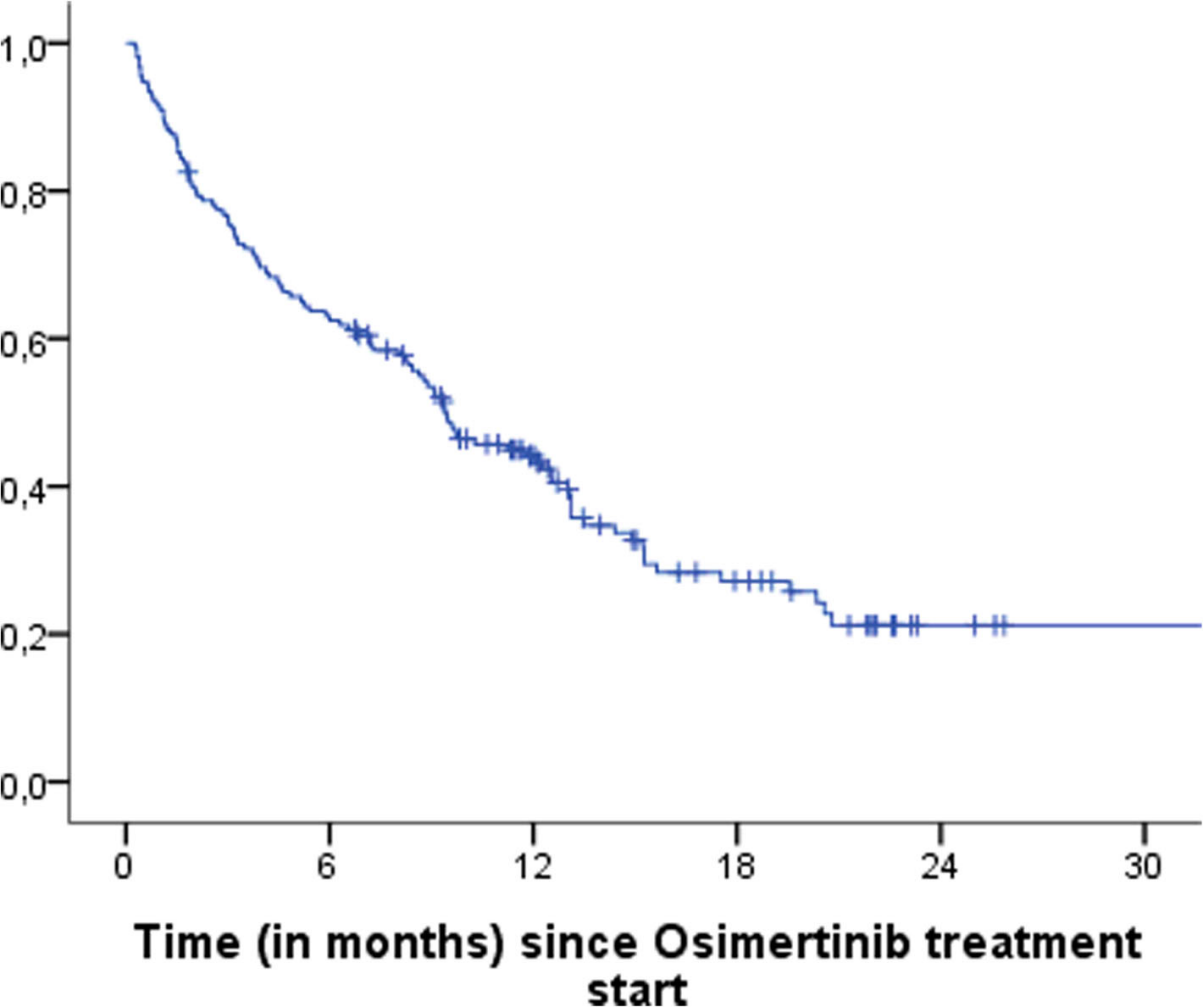
Estimated median PFS of patients treated with osimertinib

Of the 155 patients included, 89 (57.4%) progressed during follow up, 16 (10.3%) died before progression and 50 (32.3%) were still alive without progression at the trial’s closure. Figure 2 shows the estimated median PFS curve for this type of patients, with a median PFS of 9.4 months (95% CI, 7.3–11.6 months).

PFS can be compared by treatment line. Of the 87 patients that received osimertinib as first or second line, 61 progressed or died during follow up (70.1%). Of the 68 patients who received osimertinib as third or further line, 44 progressed or died during follow up (64.7%). No statistically significant differences were observed between the PFS curves of both groups of patients (*p*-value = 0.113).

### Follow up of patients with CNS metastases at osimertinib treatment initiation

Presence of CNS metastases was registered at the beginning of osimertinib treatment in 45 patients (29.9%).

Significant differences were observed according to the absence or presence of brain metastases prior osimertinib initiation [10.3 months (95% CI, 7.8–12.8 months) and 7.2 months (95% CI, 3.9–10.6 months), respectively (HR = 1.546 with 95% CI, 1.030–2.321); *p*-value = 0.034].

No significant differences were observed when comparing OS according to presence or absence of CNS metastases at the initiation of osimertinib treatment (p-value = 0.365). Median OS was 18.3 months (95% CI, 14.9–21.7 months) and 13.8 months (95% CI, 11.0–16.6 months) for those patients without and with brain involvement prior to osimertinib initiation, respectively.

### Follow up of patients that received osimertinib as last treatment

Overall survival for the 134 patients that received osimertinib as last treatment (no posterior treatment registered) was compared with that of the 21 patients that received subsequent chemotherapy and no significant differences were observed (p-value = 0.411).

Of the 134 patients, whose last treatment was osimertinib, the end date was not registered for 52 of them, so they continued on treatment at the date of treatment data collection closure (31/12/2018).

### Follow up of T790M+ “de novo” patients

Overall survival and PFS of the 10 (8.3%) “T790M+ de novo” patients were analyzed.

The small number of these subgroup of patients does not lend itself to precise estimates, making it difficult to detect potential differences. Estimated median OS for these patients was 14.8 months (95% CI, 3.1–26.5 months), while estimated median PFS was 8.6 months (95% CI, 0.0–18.1 months).

Of the 6 patients included in this group that had received previous treatment, 3 (50%) died during follow up. Estimated median OS for these patients was 15.6 months, while estimated median PFS was 9.4 months.

### Health service resources

Data on use of health service resources during treatment with osimertinib (from first dose to last) was collected. It was assumed that all 155 patients had finished treatment with osimertinib, so none were censored.

The use of health service resources is described according to previous characteristics before osimertinib treatment. Table 4 shows the main number of resources used according to different characteristics. A comparison between the values observed using the Mann-Whitney U test is also shown. The results show that stage T3-T4 patients consumed more resources, with a significant difference in R13 (other imaging tests). Similarly, non-smokers and those who had more previous chemotherapy lines also required more resources.

**Table 4:** Health service resource use during osimertinib treatment

	Sum	Main	DT	Median	Minim	Maxim
**Patients**						
**HOSPITAL CARE health resources (*n*** **= 155)**						
R01: Hospital visits/admissions (number)	1421	9.2	14.0	1	0	73
R02: Visits to the Medical Oncologist (number)	2011	13.0	10.0	11	0	56
R03: Visits to other hospital Physician (number)	478	3.1	5.5	1	0	48
R04: Visits to the Hospital Nurse (number)	560	3.6	6.5	0	0	39
R05: Emergency room visits (without admission) (n.)	143	0.9	1.6	0	0	9
R06: Hospitalization in the emergency room (days)	54	0.3	1.0	0	0	6
R07: Hospitalization in ward (days)	759	4.9	14.6	0	0	133
**IMAGING TESTS (*n*** **= 155)**						
R08: Chest x-ray (number)	333	2.1	3.1	1	0	16
R09: CT scan (number)	475	3.1	2.5	3	0	12
R10: Magnetic resonance (number)	113	0.7	1.3	0	0	6
R11: Endobronchial ultrasound (number)	2	0.0	0.2	0	0	2
R12: Esophageal Endoscopic Ultrasound (number)	2	0.0	0.2	0	0	2
R13: Other tests (number)	65	0.4	1.2	0	0	10
**OTHER complementary tests (*n*** **= 155)**						
R14: Bone scintigraphy (number)	32	0.2	0.6	0	0	3
R15: Sputum cytology (number)	1	0.0	0.1	0	0	1
R16: Thoracentesis (number)	12	0.1	0.3	0	0	3
R17: Biopsy (number)	64	0.4	1.5	0	0	9
R18: Bronchoscopy (number)	12	0.1	0.4	0	0	4
R19: Mediastinoscopy (number)	0	–	–	–	–	–
R20: Mediastinotomy (number)	0	–	–	–	–	–
R21: Thoracoscopy (number)	0	–	–	–	–	–
R22: Spirometry (number)	5	0.0	0.2	0	0	1
R23: Plethysmography (number)	0	–	–	–	–	–
R24: Electrocardiogram (number)	198	1.3	3.6	0	0	30
R25: Other tests (number)	18	0.1	0.4	0	0	4
**PRIMARY CARE health resources (*n*** **= 152)**						
R26: Visits to primary care (number)	271	1.8	5.4	0	0	39
R27: Visits to primary care Physician (number)	257	1.7	5.0	0	0	36
R28: Visits to primary care Nurse (number)	166	1.1	5.1	0	0	37
R29: Tests performed (number)	112	0.7	4.8	0	0	50
R30: Chest x-ray (number)	20	0.1	0.9	0	0	10
R31: Complete blood count (number)	224	1.5	6.0	0	0	50
R32: Other tests (number)	6	0.0	0.3	0	0	3
**LABORATORY TESTS (*n*** **= 155)**						
R33: Immunohistochemistry (number)	35	0.2	1.3	0	0	15
R34: Molecular tests (EGFR, ALK, ROS1, BRAF) (num.)	66	0.4	1.0	0	0	5
R35: Complete blood count (number)	1.873	12.1	10.5	9	0	62
R36: Blood chemistry (number)	1.859	12.0	10.4	9	0	62
R37: Proteins in urine (number)	310	2.0	6.3	0	0	35
R38: Other tests (number)	119	0.8	3.2	0	0	29
**SURGERIES (*n*** **= 155)**						
R39: Pneumectomy (number)	0	–	–	–	–	–
R40: Lobectomy (number)	0	–	–	–	–	–
R41: Segmentation or wedge resection (number)	0	–	–	–	–	–
R42: Sleeve resection (number)	0	–	–	–	–	–
R43: Others (number)	5	0.0	0.2	0	0	2
**OTHER RESOURCES/SUPPORT (*n*** **= 155)**						
R44: Radiofrequency ablation (number)	1	0.0	0.1	0	0	1
R45: Radiotherapy palliative (number of sessions)	159	1.0	4.0	0	0	40
R46: Pleurodesis (number)	1	0.0	0.1	0	0	1
R47: Corticosteroids	no	117	75.5%	yes	38	24.5%
R48: Bisphosphonates	no	142	91.6%	yes	13	8.4%
R49: Stimulants of erythropoietin	no	151	97.4%	yes	4	2.6%
R50: Blood transfusions	no	147	94.8%	yes	8	5.2%
R51: Enteral nutrition	no	153	98.7%	yes	2	1.3%
R52: Others	no	145	93.5%	yes	10	6.5%

## Discussion

Obtaining real-life data is a very important factor in understanding drug efficacy. Typically, clinical trials include a highly selected population that is not always representative of real-world clinical practice. Our study consolidates information about the efficacy of osimertinib in any line of treatment, thus over 40% of our patients received the drug beyond third line, and in 78% of cases patients had some type of comorbidity.

In fact, PFS was 9.4 months (95% CI, 7.5–11.6), which does not show significant differences even in fourth line, according to the literature [13].

It should be remembered that standard treatment with chemotherapy in these patients does not reach more than 5–6 months PFS, at best [14]. Other real-world studies with osimertinib [15] have reached PFS of 10.1 months (95% CI, 9.2–11.0 months), including a recently published pooled analysis [16] with median PFS of 9.9 months (95% CI, 9.5–12.3), very similar to our own.

Similarly, for the other objective measure of efficacy, ORR, significant differences were not observed by treatment line. Our study obtained ORR of 50%, very similar to other retrospective studies [17] and to pivotal studies with osimertinib in these patients, bearing in mind they are mostly Caucasian, and Asian populations have higher response rates and, usually, longer PFS [18].

As well as efficacy, one of the most important factors is the excellent tolerance and minimal side effects with osimertinib. Of 155 patients that received treatment and were included in the study, 76 (49.0%) did not experience AEs. Only 2 (1.3%) had AEs with a maximum grade of 4, 2 patients (1.3%) had AEs with a maximum grade of 5, and discontinuation due to toxicity was just 2.6%, in line with the pivotal clinical trials published.

CNS involvement, both at time of initial diagnosis and at disease progression, is common in these patients [19] and is an indicator of poor prognosis. It should be noted that among patients with brain metastases at the start of osimertinib treatment, the majority had been previously treated (58%) and, despite this, response rate with osimertinib was 40%—rising to almost 70% if we include stabilizations—confirming the drug’s activity in this setting [8]. Notably, all this was achieved outside the framework of a clinical trial in which only asymptomatic patients with stable disease and not requiring steroids were included in the previous 4 weeks [20].

Since the publication of the results of the FLAURA study, which demonstrated clearly improved median PFS in patients treated with osimertinib (18.9 months) versus first-generation TKIs (10.2 months) [21], it seems that the debate about the role of osimertinib now no longer focuses on its activity in second lines, when the patient develops the resistance mutation, but on the treatment sequence.

It is important to note that the greatest use of resources occurs when more lines of treatment have been given prior to osimertinib. Therefore, in addition to the clear benefit of early-line osimertinib, toxicity is reduced and the economic benefit is increased.

## Conclusion

Given all of the above, we believe that it is important to report real-world data from patients treated with osimertinib in any line. Our results with 155 patients are similar to those of real-world studies in China with 77 patients [22], Germany with 51 [15], or France with 205 patients [23] and demonstrate that real-world data closely agrees with that obtained in pivotal clinical trials, even without patient selection.

## Data Availability

All data generated or analyzed during this study are included in this published article.

## Abbreviations

AEs: Adverse events
CNS: Central nervous system
CRF: Case report form
DOR: Duration of response
EPA-OD: Post-authorization study, other designs
IEC: Independent ethics committee
NSCLC: Non-small cell lung cancer
ORR: Objective response rate
OS: Overall survival
PFS: Progression-free survival
PR: Partial response
RDC: Remote data capture system
SD: Stable disease
SLCG: Spanish lung cancer group
SUMP: Special use medication program
TKI: Tyrosine kinase inhibitors
